# Physicians' Perceptions of Information Quality and Access in a New Cross-Institutional Electronic Health Record (EHR) System: Mixed Methods Design

**DOI:** 10.2196/90797

**Published:** 2026-10-20

**Authors:** Olga Golburean, Line Melby, Rune Pedersen, Signe Lohmann-Lafrenz, Sigmund Østgård Gismervik, Eivind Schjelderup Skarpsno, Arild Faxvaag

**Affiliations:** 1 Department of Neuromedicine and Movement Science, Faculty of Medicine and Health Sciences, Norwegian University of Science and Technology, Elgeseter Gate 28, Trondheim, Trøndelag, 7030, Norway, 47 91787389; 2 Department of Dental Hygiene, Faculty of Dentistry, University of Oslo, Oslo, Norway; 3 Department of Health Research, SINTEF Digital, Trondheim, Norway; 4 Department of Health Sciences, Faculty of Medicine and Health Sciences, Norwegian University of Science and Technology, Trondheim, Trøndelag, Norway; 5 Norwegian Centre for E-Health Research, University Hospital of North Norway, Tromsø, Norway; 6 Department of Health Sciences, Faculty of Nursing and Health Sciences, Nord University, Bodø, Nordland, Norway; 7 Department of Public Health and Nursing, Norwegian University of Science and Technology, Trondheim, Trøndelag, Norway; 8 Clinic of Thoracic and Occupational Medicine and Orkdal Dept. of Internal Medicine, St Olav's University Hospital, Trondheim, Trøndelag, Norway; 9 Clinic of Rehabilitation, St Olav's University Hospital, Trondheim, Trøndelag, Norway; 10 Helseplattformen AS, Trondheim, Trøndelag, Norway

**Keywords:** access to data, cognitive load, decision-making, electronic health record, EHR, information overload, mixed methods design

## Abstract

**Background:**

Physicians rely on electronic health record (EHR) systems to make informed decisions and deliver high-quality care. Although the introduction of EHR systems has created significant opportunities for improving patient care, it has also introduced challenges related to information overload and increased cognitive demands.

**Objective:**

This study aimed to assess physicians’ perceptions of a newly implemented cross-institutional EHR system regarding (1) the quality, access, and presentation of patient data and (2) the EHR’s effectiveness at supporting cooperation and communication with patients.

**Methods:**

This study used a cross-sectional mixed methods descriptive research design. Data were gathered through a closed-ended questionnaire, participant observations, and interviews with physicians at a university hospital in central Norway, which transitioned to a new EHR system in November 2022. Qualitative data were gathered from September 2023 to January 2024 and analyzed using reflexive thematic analysis, while quantitative data were collected from January 2024 to July 2024 and analyzed using descriptive statistics.

**Results:**

The quantitative data showed that most surgical and nonsurgical physicians *disagreed* on the following: (1) that the patient’s current medication list is presented in a clear format, (2) that the system provides a comprehensive summary view of patients’ health status, and (3) that the system supports cooperation and communications with patients. These results were supported by the qualitative data, indicating that the physicians stressed the necessity of improving the presentation and organization of data and voiced concerns about the overwhelming volume of information. However, physicians also emphasized the benefits of enhanced access to patient data, the usefulness of the timeline function for monitoring hospitalized patients, and the efficiency of communication among health care professionals through the chat function. Additionally, some informants reported favorable experiences with the patient health portal, which facilitated more efficient communication with patients.

**Conclusions:**

This study revealed a complex landscape regarding physicians’ experiences with the newly implemented cross-institutional EHR system. Physicians stressed the need for improved organization and presentation of patient health data while also highlighting the positive impact of a cross-institutional EHR system on enhanced access to data. Although quantitative data showed that most physicians disagreed on whether the new EHR enhances cooperation and communication with patients, some informants in the qualitative part of the study highlighted positive experiences related to the patient health portal in improving communication. Based on qualitative findings, adding a feature that allows users to confirm if the patient has received or read messages could further improve the usefulness of the patient portal. Our qualitative findings also emphasized that enhancing system usability must be accompanied by comprehensive training on EHR use to achieve meaningful use. Future evaluations of the EHR system should focus on examining its long-term effects on access to patient data.

## Introduction

### Background

In many jurisdictions, a patient is defined as an individual who seeks assistance from a health care professional regarding a health problem. By seeking help, the patient implicitly entrusts the health care professional with a mandate for their care. In accepting the care mandate, the health professional accepts being held accountable for the decisions made, which begins with gathering relevant information, such as looking up the patient’s medical history and laboratory results as well as interviewing and examining the patient [1]. The decisions are based on both the available information and the health professional’s clinical expertise and knowledge [2]. Accurate and timely information is critical for effective diagnosis [3], treatment planning [4], and the reduction of unnecessary medical tests and interventions. Since health care professionals depend on the integrity of patient data to make clinical decisions, any shortcomings in data quality, ineffective communication, and insufficient information sharing among health care professionals can lead to adverse patient outcomes and increased health care costs [5,6].

Health care institutions should provide their professionals with an effective electronic health record (EHR) system [7]. Such a system is essential for delivering clear and concise information about patients, ensuring that health care professionals have access to the crucial data needed for informed decision-making [7]. Simultaneously, health care professionals have a duty to maintain accurate records of the care they provide [8,9]. This responsibility necessitates diligent documentation practices, as the quality of the data entered into the health record system is directly influenced by these practices [10,11]. The advent of EHR systems has transformed such information-gathering and documentation strategies, thereby altering the representation and meaning of patient data [12]. EHR systems offer timely access to comprehensive patient data acquired from different sources. These data can be accessed remotely and simultaneously by multiple health care professionals [13,14], thereby improving the efficiency and timeliness of medical decision-making [15,16]. However, the implementation of new EHR systems can also lead to information overload, poorly designed information displays, and fragmentation of data across various sections of the EHR system [17,18]. Such challenges may increase cognitive demand on health care professionals, particularly in locating and retrieving essential patient information [17-20].

As health information systems (HISs) continue to evolve in response to technological advancements, regulatory changes, and the evolving needs of health care professionals, it is important to understand physicians’ experiences and perceptions regarding the quality and access to patient health data. Such insights are crucial for guiding the design and implementation of EHR systems that meet user needs. Thus, this study aimed to assess hospital physicians’ perceptions of a newly implemented EHR system regarding (1) the quality, access, and presentation of patient health data and (2) the EHR system’s effectiveness at supporting cooperation and communication with patients.

### Theoretical Perspectives

The health care system is a complex, knowledge-driven landscape shaped by the dynamic interactions of various stakeholders [21,22]. The adoption of HISs, such as EHR systems, is primarily driven by the necessity for seamless integration of health information across various disciplines, locations, and time frames. To achieve this, a substantial body of research has explored the role of standardization in the context of HISs [23-26]. Standardization in health care is, however, often viewed as a “double-edged sword*”* [27]. On one hand, it has the potential to enhance efficiency by reducing unnecessary variation and errors [28-30]. On the other hand, it may lead to adverse consequences, such as reduced flexibility and adaptability in response to the diverse and complex nature of health care [28]. Consequently, the significance of flexibility within technical systems has become increasingly evident. For instance, Cherns [31] pointed out that these systems must be tailored to meet particular local needs and be capable of evolving in response to shifting demands over time. Moreover, Orlikowski [32] highlighted that technology goes beyond being just a tool used by organizations; rather, it is continuously influenced and redefined through ongoing human interactions. In light of these complexities, many researchers advocate for sociotechnical theories and models in the evaluation and implementation of HIS [33,34]. Sittig et al [34] stated that “socio*-*technical approaches posit that technologies operate within social and organizational contexts that are inseparable from the presence or influence of the technologies themselves.” This paper used a sociotechnical perspective to analyze the study findings. By exploring both social and technological dimensions, we can gain a deeper understanding of factors that influence physicians’ perceptions regarding the new EHR system.

## Methods

### Ethical Considerations

This study was registered and approved by Sikt–Norwegian Agency for Shared Services in Education and Research (project number 540255) and the Regional Committee for Ethics in Medical and Health Research Ethics in Central Norway (REK Central; project number 228249). Sikt is a public administrative body under the Ministry of Education and Research. Before observing physicians' interaction with the EHR system during routine clinical work, including situations involving patient interactions, patients were informed about the study and provided verbal consent for the researcher to be present. No patient data nor personally identifiable information were collected during the observations. Observation notes, interview records, and transcripts were stored securely using the NICE-1 secure storage system, which is accessed through a VPN with 2-factor authentication. When observation notes and interview transcripts were shared with the research team, they were transferred using the encrypted file transfer option in FileSender, a secure file transfer service provided by the Norwegian Agency for Shared Services in Education and Research. All participants were provided with information about the study, and written consent was obtained from each participant. Participants did not receive any financial or other compensation.

### Study Design

This study used a cross-sectional mixed methods descriptive research design with sequential data collection. The study combined qualitative and quantitative data collection methods, including participatory observations, semistructured interviews, and a survey. Observations were conducted first, followed by interviews and subsequently the survey. The sequence and timing of data collection are presented in Figure 1. The mix of quantitative and qualitative methods was chosen as it can give a broader and deeper perspective than either method could achieve independently [35,36]. The study was reported in accordance with the GRAMMS (Good Reporting of A Mixed Methods Study) [37], SRQR (Standards for Reporting Qualitative Research) [38], and STROBE (Strengthening the Reporting of Observational Studies in Epidemiology) [39] reporting guidelines. Complete checklists are available in Checklist 1.

**Figure 1.**
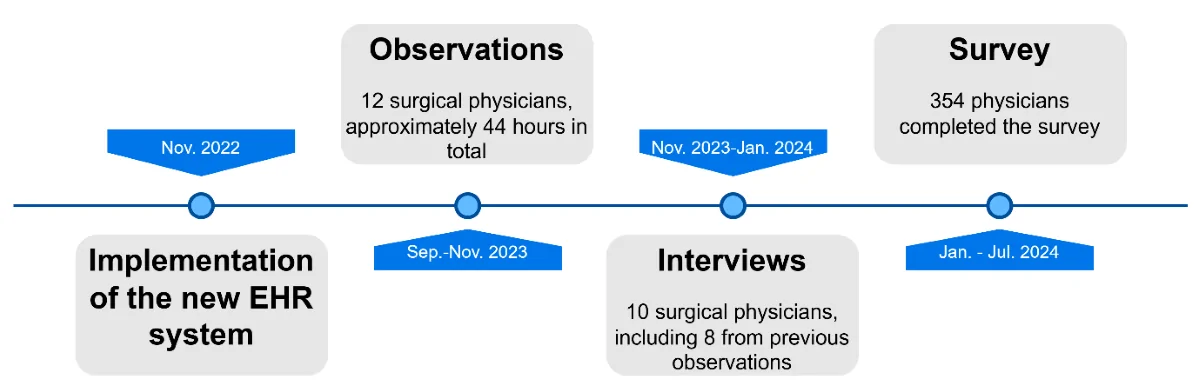
Data collection process. EHR: electronic health record.

The survey was used to examine whether the perceptions identified in the qualitative part of the study were also reflected among a broader physician population. Thus, the qualitative and quantitative components were designed to be complementary, with the qualitative findings providing contextual depth and the survey providing an opportunity to assess whether the patterns identified qualitatively were also reflected in a larger and more diverse sample of physicians. To facilitate comparison between the 2 datasets, all survey questions included in this study were also incorporated into the interview guide, though they were explored in greater depth during the interviews through follow-up questions. Priority was given to the qualitative component because the primary aim was to gain an in-depth understanding of physicians’ experiences. It is important to note that the qualitative sample consisted exclusively of surgical physicians, whereas the survey included both surgical and nonsurgical physicians. Consequently, direct comparison between the qualitative and quantitative findings was limited to topics relevant to surgical physicians. The qualitative findings should therefore be interpreted as providing contextual understanding of surgical physicians rather than as representative of all physicians included in the survey. Findings from the nonsurgical respondents were used to assess whether similar quantitative patterns were observed across other physician groups. The qualitative and quantitative data were analyzed independently, with integration occurring during the interpretation. Following the approach described by Guetterman et al [40], the findings from the 2 components were integrated and interpreted through narrative discussion and a joint display.

The interviews and observations were collected as part of a broader study aimed at addressing 2 distinct research questions. A previous publication reported findings on physicians’ documentation practices following the implementation of the new EHR system [41], whereas this paper reports findings on physicians’ perceptions regarding patient data within the system, as outlined in the Introduction section.

### Study Settings and Context

This study was conducted with physicians at a large university hospital in central Norway, which in November 2022, transitioned from a facility-centered system to a new cross-institutional EHR system. Deployment of the new EHR project is part of the Norwegian government’s “One Citizen-One Health Record” initiative, which aimed to replace all existing EHRs in central Norway with one integrated EHR system for municipalities, hospitals, general practitioners, and specialist health care professionals [42]. The new EHR system is anticipated to enhance access to patient data for health care professionals at the point of care by standardizing clinical data and improving interoperability across various health care organizations. Epic Systems Corporation was selected as the vendor for the EHR system. This marks the largest-scale implementation of an integrated EHR system in Norway [43] covering 34 municipalities, 9 hospitals, 2 clinics, and 2 emergency departments across central Norway [44]. In addition, the new EHR system integrates with about 200 other systems via Epic’s open APIs, thus enhancing information exchange [44]. Nevertheless, despite the anticipated benefits, such as easier access to patient information [45] and enhanced patient safety through digital support for correct and secure medication handling [45], the initial deployment at the university hospital revealed several challenges. This includes, as noted by the Norwegian Board of Health Supervision (Helsetilsynet), a potential for errors that could compromise patient safety, usability issues, and workflow disruptions [46]. Although some improvements have been made, and Helsetilsynet, in a later release, outlined that the level of risk for the patients was effectively reduced, ongoing challenges remain and still require additional time and effort to address [47]. The new EHR system is significantly different from the previous system (DocuLive), which was a hospital facility-centered electronic patient record (EPR). DocuLive was primarily a free-text journal, with limited structured and standardized documentation and limited standardized pathways [48]. On the other hand, it allowed health care professionals greater flexibility. As a result, the new EHR system required health care professionals to make significant adjustments, as they needed to become familiar with and learn how to use a complex new system, record patient data using structured and standardized formats, and adapt to a different presentation of patient information than what they were used to in DocuLive [41,45]. Additionally, the implementation of the new EHR system received substantial media critique, which may further influence health care professionals’ perceptions of the system.

### Qualitative Data Collection and Study Population

The qualitative part of the study included participant observations and semistructured interviews with surgical physicians at the University Hospital in central Norway. We chose to focus on the surgical department due to its engagement in critical decision-making processes under stringent time constraints, which require prompt and accurate judgments facilitated by prompt access to patient data [49]. In this context, the impacts of the new EHR system were expected to appear shortly after its implementation, particularly concerning the anticipated advantages related to real-time access to patient information at the point of care, primarily attributed to the cross-institutional design of the new EHR system and its emphasis on interoperability [42,44]. Focusing on a single department within this qualitative part of the study was a deliberate decision. This approach was chosen because focusing on various specialties could have resulted in a large and highly diverse dataset, which, as noted by Malterud [50], could complicate the interpretation of results and diminish the depth of the data collected. A further rationale for including only one surgical department is its wide scope, comprising various types of units, including the outpatient clinic, inpatient ward, emergency room, surgical unit, and pediatric division. This offered a comprehensive view of surgical activities across multiple contexts.

Qualitative data were collected from September 2023 to January 2024, approximately 10 months to 13 months following the implementation of the new EHR system. Participants were recruited through written invitations (email) and verbal approaches. Initially, all physicians within the surgical department were invited to participate in an observational study. However, the initial recruitment process proved challenging owing to physicians’ heavy workloads and the new EHR system introduction. To address this, the researcher (OG) attended a morning meeting of the physicians’ morning meetings, provided additional information about the study, and allowed physicians to ask specific questions directly. Interested physicians then contacted the researcher (OG) to arrange observation sessions. After the observations, the same group of physicians was invited via email to participate in semistructured interviews. In total, 12 physicians were observed for about 44 hours, and 10 participated in individual, face-to-face interviews, with 8 having been previously observed. Refer to Figure 1 for the data collection process.

During the observation sessions, the researcher (OG) observed surgical physicians as they used the new EHR system during their typical workday both in outpatient clinics and inpatient wards. Throughout each session, OG took detailed notes and, when appropriate, engaged in discussions with the surgical physicians about their use of the new EHR system, including aspects such as access, quality, and presentation of patient data. Any further questions that arose during the observation were addressed with the surgical physicians immediately after each session. Additionally, when time allowed, physicians occasionally demonstrated how patient data were displayed and the steps needed to access it in the EHR system.

Observational sessions were conducted prior to the interviews to gain a deeper understanding of the clinical context and how physicians interacted with and adapted to the new EHR system. As highlighted by Berg [51], observations provide insights into actual work practices and system use, complementing interview data by capturing behaviors and interactions that participants may not explicitly describe during the interviews. Because many of the observed physicians were subsequently interviewed, the observations enabled us to ask more specific, contextually grounded questions about the situations we had observed. In addition, the combination of observational and interview data enabled triangulation of findings, providing a cross-validation of insights from different data sources and thereby strengthening the credibility of the study [52].

The individual interviews were conducted by OG and ranged from 34 minutes to 60 minutes in length, with an average duration of 44 minutes. The interview guide was developed based on existing literature [53-55] and input from field experts (OG, AF, LM, and RP). It also included 3 questions from the quantitative questionnaire used in the study (Multimedia Appendix 1). Each interview was audio-recorded and transcribed verbatim by the first author (OG).

### Qualitative Data Analyses

The interview transcripts and the field notes from the observation sessions were organized with the help of Nvivo software [56] and were analyzed using a reflexive thematic analysis method [57]. The interview transcripts and field notes from the observations were initially read in order to familiarize oneself more thoroughly with the dataset. The initial codes were then developed using an inductive semantic coding method and assessed by the first author (OG) to establish initial themes. To ensure trustworthiness, peer debriefings, which include seeking feedback from other researchers, were used [58-60]. Initially, the authors (OG, AF, LM, and RP) were provided with randomly selected field notes and interview transcripts to facilitate their familiarization with the raw data. After they were also familiarized with the data, several workshops were held in which the authors (OG, AF, LM, and RP) collaboratively reviewed preliminary themes and codes. These authors were included in the workshop, as they were also involved in planning the qualitative part of the study. During this stage, a latent analytic approach was used, which shifted focus from descriptive accounts to the underlying patterns and meanings in the data [61]. The data interpretation was guided by the sociotechnical theory [34,62] to examine how the interaction between technical and human factors influenced the experiences of surgical physicians with the new EHR system; the interpretations were further refined through ongoing discussions among researchers, drawing on collective expertise in health informatics and EHR evaluation. Following this collaborative effort, OG further refined the themes, and the adjustments were continuously deliberated with the authors, with disagreements being resolved during these discussions.

### Reflexivity

The first author (OG), PhD candidate in Medical Technology at the Norwegian University of Science and Technology (NTNU), responsible for qualitative data collection and initial analysis, had no prior relationship with the participants. Through her PhD research and active involvement in the Nordic eHealth Research Network (NeRN), as well as participation in workshops and meetings with Norwegian health authorities and World Health Organization (WHO) Europe, she had developed substantial knowledge of HISs, EHR implementation, and digital transformation in health care. This background provided both methodological and contextual expertise that informed data collection and interpretation of the findings. The coauthors (AF, RP, and LM) are professors with extensive experience in health informatics and EHR system evaluation, with LM and RP possessing extensive expertise in qualitative research methods. They contributed to the study design and were actively involved throughout the analytic process, allowing different perspectives and experiences to inform the analysis.

### Quantitative Data Collection and Study Population

Quantitative data were collected from January 2024 to July 2024 through a web-based questionnaire that was distributed via email to all health professionals employed at the hospital during the data collection period, including residents. Because all physicians were invited to participate, no formal sample size calculation was performed. Duplicate responses were prevented through the use of unique survey links and participant identifiers. In total, the questionnaires were sent to 1317 physicians. Only physicians who reported using the EHR system and completed the relevant National Usability-Focused Health Information System—Scale (NuHISS) items were eligible for inclusion in these analyses. In total, 354 (354/1317, 26.9%) participants completed the NuHISS items (71 from the surgical specialties and 283 from the nonsurgical specialties). The surgical specialties encompassed breast and endocrine surgery, gastrointestinal surgery, gynecological surgery, neurosurgery, orthopedic surgery, plastic surgery, thoracic surgery, urology, and more. The questionnaire used in this study was adapted from the validated Finnish NuHISS questionnaire [63]. The questionnaire comprised 23 questions; however, for the purposes of this study, we focused our analysis on 3 questions related to the information quality: (1) The patient’s current medication list is presented in a clear format*,* (2) The EHR system generates a summary view (eg, on a timeline) that helps to develop an overall picture of the patients’ health status, and (3) EHR systems support co-operation and communication between clinicians and patients. The choice to focus on the information quality dimension was guided by the study’s aim. It is important to note that the information quality dimension of the Norwegian version of NuHISS is based on a smaller set of items (3 questions rather then the 5 questions included in the original Finnish instrument). The researchers from the Norwegian National Center for eHealth Research, in collaboration with the Nordic NuHISS group and the NeRN, decided to reduce the number of questions to ensure cross-national comparability and relevance in the Norwegian context. Additionally, other dimensions such as “ease of use,” which capture conceptually distinct aspects of system usability, were considered outside the scope of this analysis. Nevertheless, the remaining dimensions and items from the Norwegian NuHISS questionnaire that were not included in this study will be analyzed and reported separately as part of the broader STUNTH (The Study of New Technology and Health among Hospital employees) study [64].

The questions were rated using a 5-point Likert scale, ranging from strongly agree to strongly disagree (Multimedia Appendix 2). Participants who answered “strongly agree” or “agree” were categorized as “agree,” and those who answered “disagree” or “strongly disagree” were categorized as “disagree.”

### Statistical Analysis

Descriptive statistics of the study sample were performed and reported in terms of frequencies and percentages for categorical variables and means and standard deviations for continuous variables. Data from the 3 NuHISS questions on information quality are presented in bar charts with percentages and 95% CIs. Missing data were handled using complete-case analysis for each item, with item-specific denominators reported. No imputation of missing values was performed. “Not relevant” responses were analyzed and reported separately rather than being treated as missing data. Quantitative analyses were performed using the statistical software STATA [65], and bar charts were generated using Microsoft Excel. To enhance transparency, row counts and item-specific denominators for all NuHISS items used in the analysis of this study are reported in Multimedia Appendix 3.

## Results

### Participants

In the quantitative part of the study, 354 of 1317 eligible participants completed the survey, yielding a response rate of 26.9%. The participants had an average age of 45 (SD 10.2) years (Table 1). In the qualitative part, which included observations and interviews, a total of 14 surgical physicians participated, with a mean age of 40 (SD 9.8) years (Table 1).

**Table 1.** Demographic profiles of respondents from the qualitative part of the study (n=14) and quantitative part of the study (n=354).

Characteristic	Qualitative part of the study, n	Quantitative part of the study, n (%)
Age group (years)^a^		
≤39	9	116 (32.8)
≥40	5	236 (66.7)
Sex^b^		
Female	6	241 (68.1)
Male	8	112 (31.6)
Clinical context		
Surgical	14	71 (20.1)
Nonsurgical	0	283 (79.9)

a Missing data for 2 participants in the quantitative part of the study.

b Missing data for 1 participant in the quantitative part of the study.

### Quantitative Research Results

#### Presentation of the Medication List

Access to the medication list serves as a representative example of access to vital patient data. Participants were surveyed about the presentation of the patient’s medication list in the new EHR system. As shown in Figure 2, a substantial majority of both surgical and nonsurgical physicians indicated dissatisfaction with the presentation of the medication list, and only approximately 10% (33/354, 9.3%) of physicians felt that the medication list was presented clearly.

**Figure 2.**
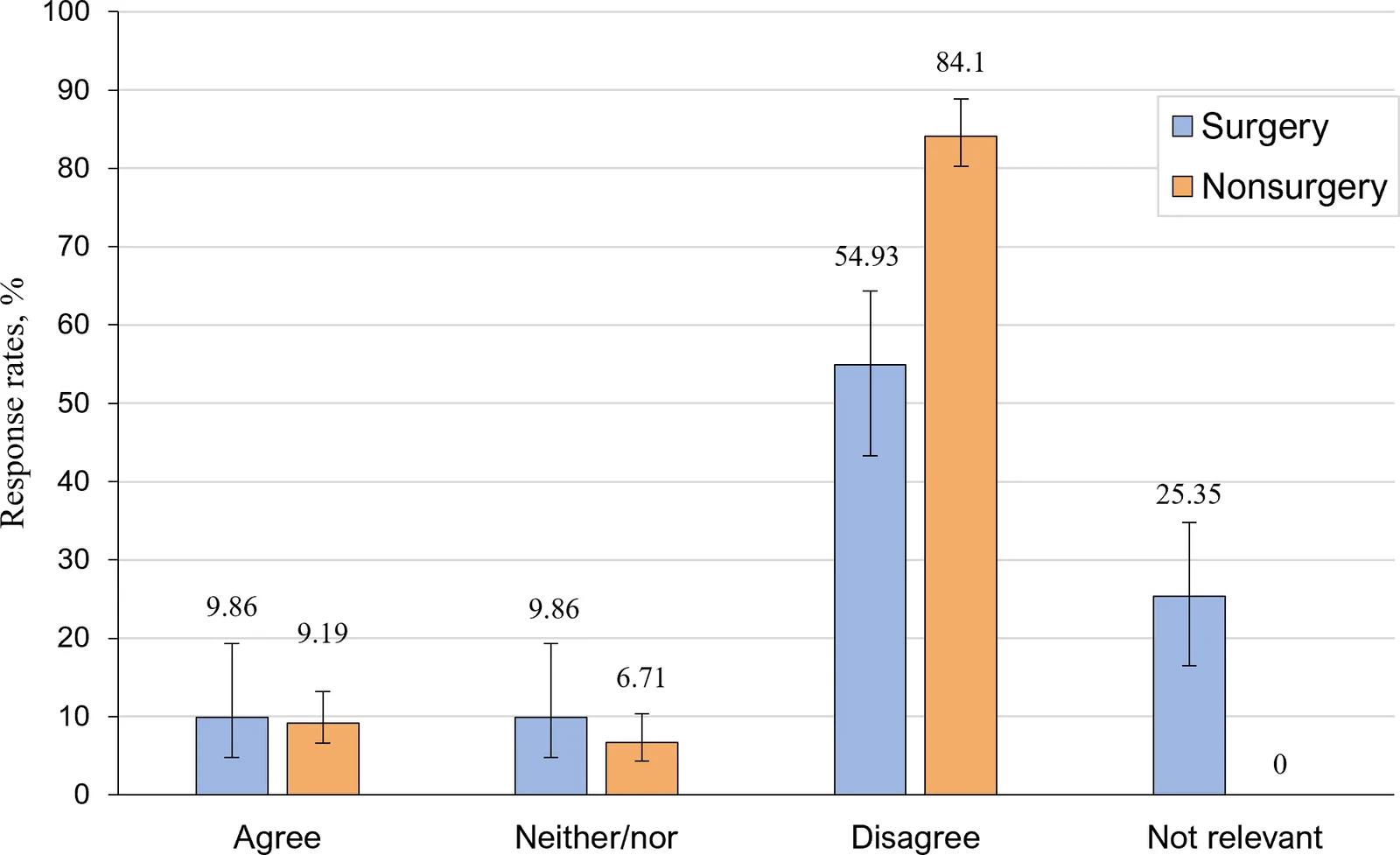
Surgical and nonsurgical physicians’ responses to “The patient’s current medication list is presented in a clear format,” expressed as percentages and 95% CIs.

#### Summary View of the Patient’s Health Status

Further, we surveyed respondents about whether the new EHR system generates a summary view (eg, on a timeline) that helps them develop an overall picture of the patient’s health status. The results revealed that only 5.6% (4/71) of surgical physicians and 16.2% (46/283) of nonsurgical physicians agreed with this statement. In contrast, a significant majority (255/354, 72%) of physicians expressed disagreement about the system’s effectiveness in this regard (Figure 3).

**Figure 3.**
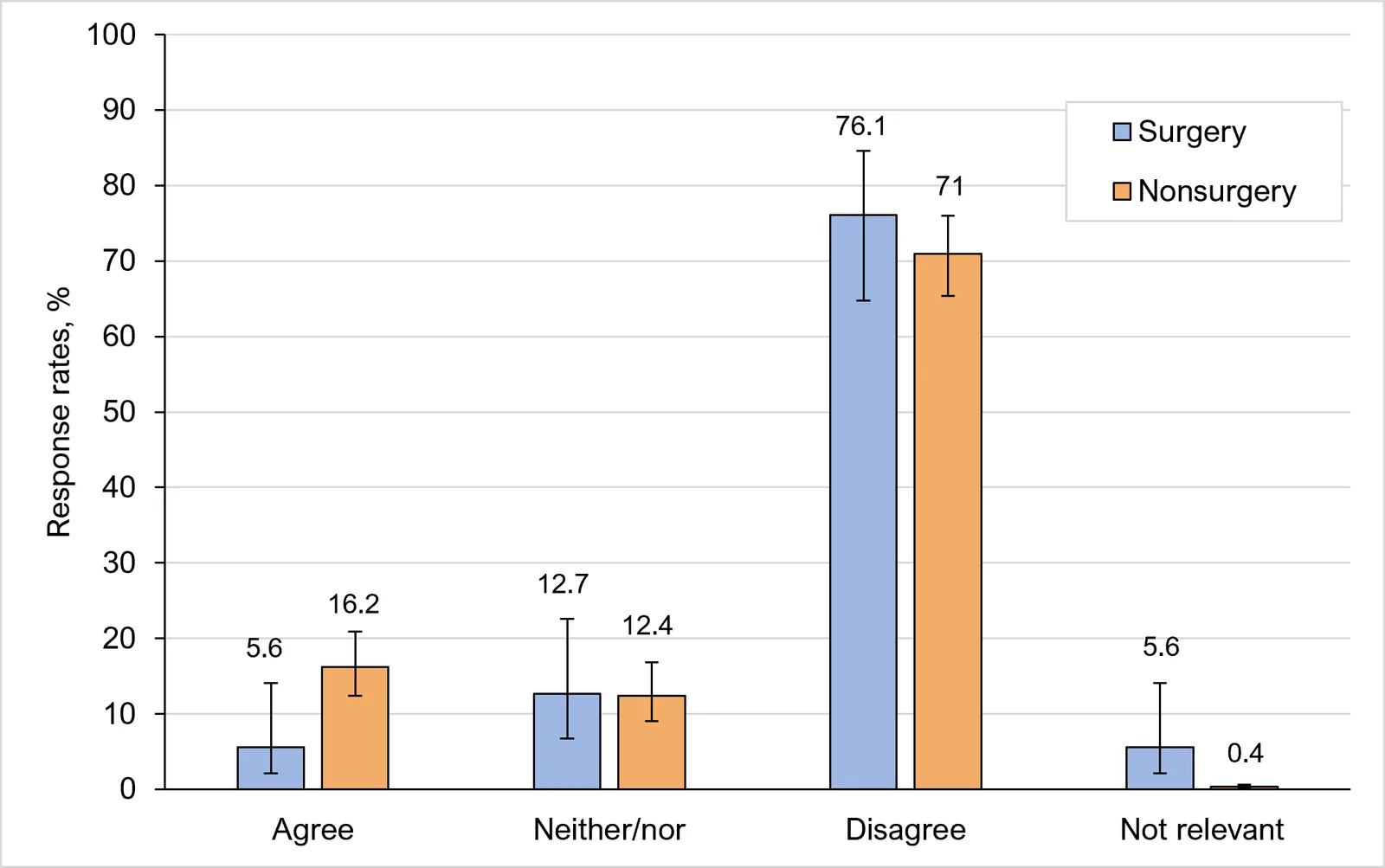
Surgical and nonsurgical physicians’ responses to “The EHR system generates a summary view that helps to develop an overall picture of the patient’s health status,” expressed as percentages and 95% CIs. EHR: electronic health record.

#### Cooperation and Communication Between Clinicians and Patients

Figure 4 illustrates the responses of physicians regarding the effectiveness of the new EHR system at supporting cooperation and communication between clinicians and patients. Among the respondents, 9.9% (7/71) of surgical physicians and 17.7% (50/283) of nonsurgical physicians agreed with the statement. In contrast, a significant percentage disagreed, representing 49.3% (35/71) of surgical physicians and 62.9% (178/283) of nonsurgical physicians.

**Figure 4.**
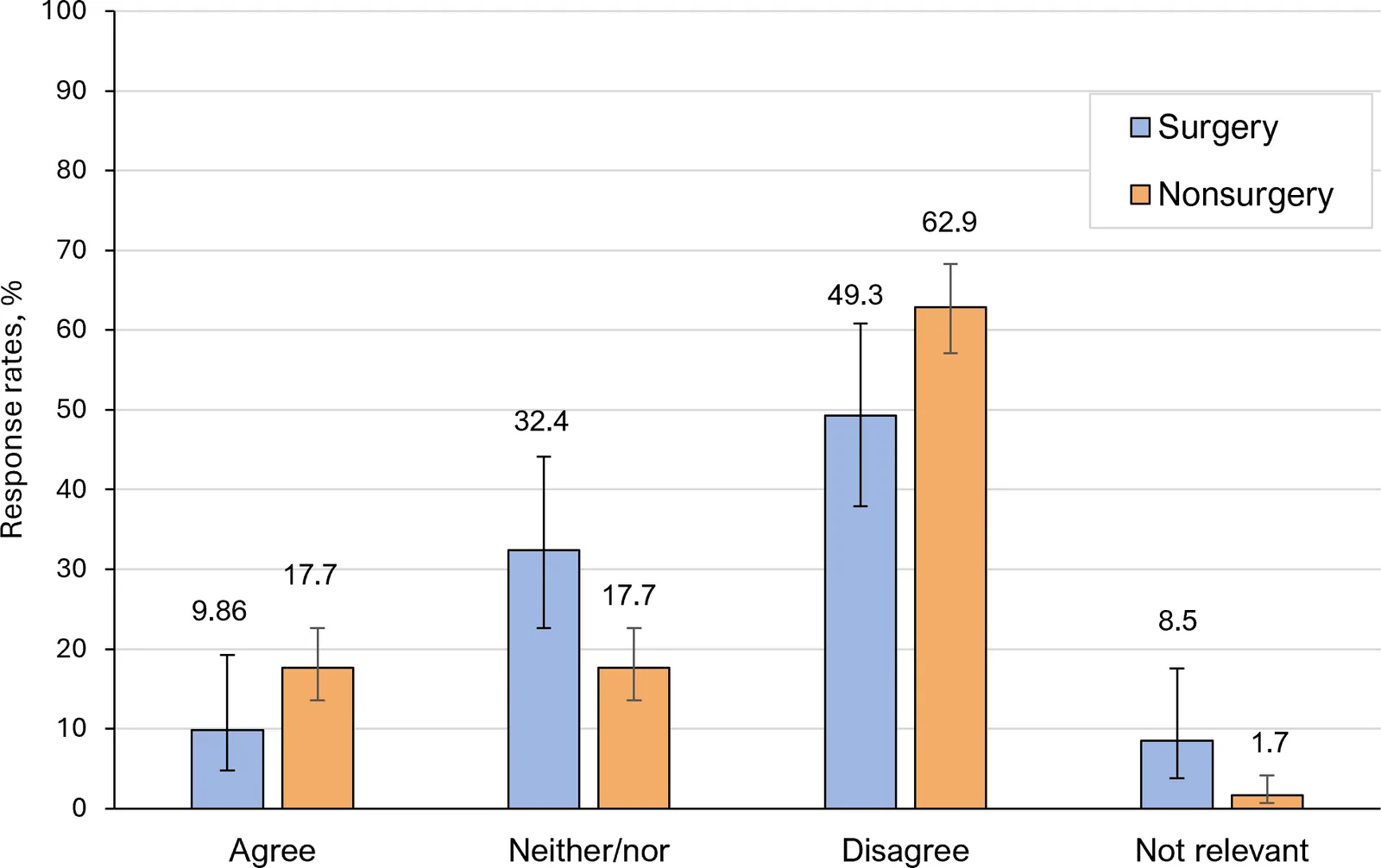
Surgical and nonsurgical physicians’ responses to “The EHR system supports cooperation and communication between clinicians and patients,” expressed as percentages and 95% CIs. EHR: electronic health record.

### Qualitative Research Results

#### Overview

This section outlines the findings from the qualitative study. The analysis revealed 2 primary themes along with their associated subthemes. See Figure 5 for the themes and subthemes identified in the analysis. Although the new EHR system presented more patient information to surgical physicians and enhanced communication within health care teams compared with previously, it also resulted in an excessive amount of information according to the informants.

**Figure 5.**
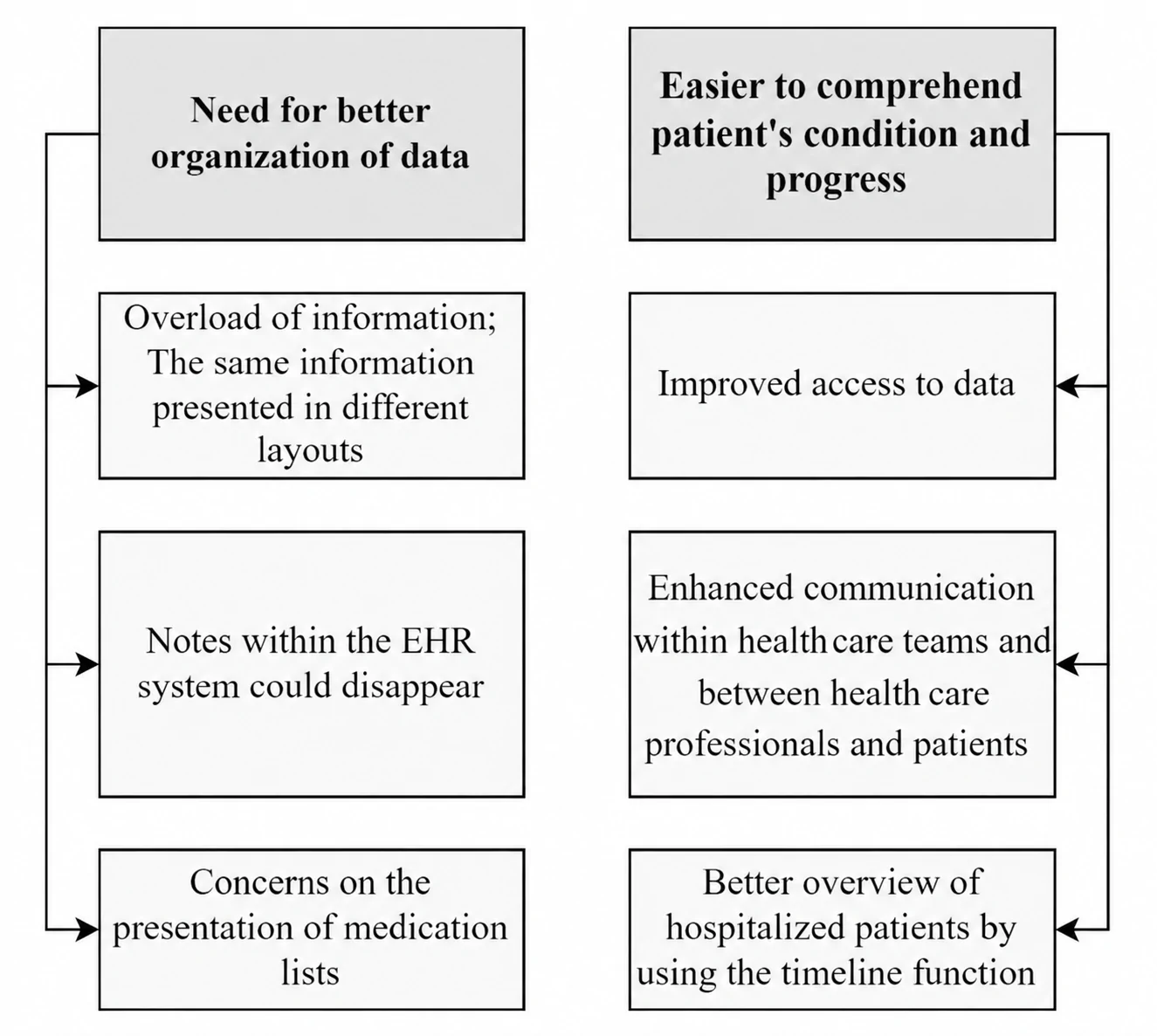
Themes and subthemes emerged from the qualitative analysis of participant observations and semistructured interviews with surgical physicians. EHR: electronic health record.

#### Theme 1: Need for Better Organization of Data

##### Overload of Information; the Same Information Presented in Different Layouts

Surgical physicians were frustrated with how the information was organized within the new EHR system. Many were overwhelmed by the very amount of information they deemed irrelevant in both outpatient clinics and inpatient wards and with the challenge of navigating multiple tabs in search of specific information. The informants also feared that critical information could be drowned out by the sheer volume of irrelevant details.

*[…] It is a lot of information that I don’t need, which is there; that might be the biggest problem. There is very, very much information that I simply do not need to see.*

[Surgical physician 4]

Many informants noted that the same information could be found at multiple locations within the system. The only difference was the layout. This could sometimes create confusion.

*Same information with a different layout, for example when it comes to lab results, you can access it through this tab called summary and journal review, and it looks a bit different, but it’s still the lab results for the patients […] and I also think that having a tab called notes and another tab called journal review, and then having a sub-tab called notes, like it is now, is very unnecessary, because you only need one place to view the notes […].*

[Surgical physician 4]

The overwhelming amount of information presented on screens could prolong the time needed to process that information. One physician remarked that transitioning to a larger screen enhanced his ability to comprehend the information displayed. Some informants also highlighted that the new EHR allowed physicians to customize or personalize certain aspects, such as screen layouts, to better align with their individual preferences. However, it was also mentioned that they neither received sufficient training nor had enough time to fully leverage these features. Moreover, some informants also remarked that the new EHR system had become easier to navigate over time, attributing this improvement to them becoming more familiar with the system. The informants also expressed a desire for a more intuitive way to access relevant information, eliminate duplicate information, and improve navigation.

*[…] with experience, you can peel away some of what you know isn’t that important, and it may be that this is why the new EHR is easier to use now than it was at the beginning because you have a better understanding of what you can overlook in a way […].*

[Surgical physician 3]

##### Notes Within the EHR System Could Disappear

Some informants experienced instances where certain notes within the EHR seemed to disappear or were difficult to locate. In one particular case, this issue resulted in the clinician having to assess the patient twice.

*I have experienced that a patient has been assessed in the emergency department from scratch by another physician because for some unknown reason they did not see my note. It hasn’t happened many times, but I particularly remember one instance where they started the evaluation of the patient from scratch again, even though my note was right next to theirs, but it was not visible to them, which was a bit strange.*

[Surgical physician 1]

The new EHR system included a filtering feature that enabled surgical physicians to sort the notes. For instance, they could choose to view only the notes written by physicians, view those recorded by nurses, or search for notes from a specific physician or department. However, observations revealed that not all surgical physicians were aware of this functionality, and some only discovered it long after the EHR had been implemented. Among those who did use the feature, opinions on its effectiveness were mixed. Although some found it highly beneficial for organizing information, others reported issues, noting that the filtering function could inadvertently obscure not only irrelevant notes but also important information they needed.

### Concerns on the Presentation of Medication Lists

Several informants highlighted the lack of clarity in medication lists. They weren’t always up to date, and in some instances, it was challenging to differentiate between medications in use before admission and those added afterward, making it time-consuming to obtain a clear overview of patients’ medications.

*[...] It does not differentiate between medications that the patient was already on prior to admission and those that were started during the hospitalization [...]. Additionally, it does not show when, who, and why medications have been initiated. I would have liked to have this presented in a chart format. I believe it is entirely possible to change that.*

[Surgical physician 4]

However, some informants also noted that, although entering and reconciling medications could be challenging, once the process was completed, the final presentation of the medication list was clear and easy to understand.

### Theme 2: Easier to Comprehend Patient’s Condition and Progress

#### Improved Access to Data

Many informants also acknowledged the benefit of increased access to information. This enhanced access allowed for a clearer overview of patients’ health statuses and facilitated better monitoring of their progress, according to some informants. Additionally, the informants noted that obtaining data from other health care organizations had become easier; for example, they had easier access to information from home health care services and emergency rooms, which had been more difficult to obtain in the previous system.

*It’s easier to find information from the municipality now because we didn’t have access to that before, so informal data from emergency rooms and home care nurses and all that, we have access to now, and that is definitely easier.*

[Surgical physician 6]

It was also noted that an important function of the new EHR system was the ability for surgical physicians to easily upload images, such as photos of a wound, directly into the patient’s medical record. This functionality was made possible by the ability to log into the EHR system from mobile devices, which enabled health care professionals to securely capture and upload images in real time. This capability was particularly valuable for tracking the progression and healing of wounds. For instance, health care professionals could take images of wounds during home visits, allowing physicians to review them when the patient arrived at the hospital.

Moreover, several informants noted that the new EHR system facilitated easier access to patient administrative data, such as waiting lists and the specific departments where patients were awaiting services. Additionally, the system included a feature for patient reminders, allowing physicians to bookmark the patients they wished to follow up.

*[…] so, there is a function set for patient reminders, where you can save patients that you want to follow up. It works fine. It was a function the old EHR didn’t have. Then, I had to write on a paper note or send a message to myself, but here there is a separate box where you can save patients you want to follow up further.”*

[Surgical physician 1]

#### Enhanced Communication Within Health Care Teams and Between Health Care Professionals and Patients

Several informants noted that communication within health care teams in the new EHR system was easier and faster because they could use the chat function. The chat function is a secure way of communication, allowing health care professionals to attach messages directly to the relevant patient’s record. These messages remained visible until addressed, which helped minimize the risk of being overlooked. The chat function reduced the reliance on phone calls and emails, enabling physicians to manage requests at their convenience.

*We have a secure chat function that is very good; you receive a message that stays there until you respond to it, so it’s not something you overlook or forget, like an email that just sits there. You can also add multiple recipients when relevant, and you can attach patient records. What’s important, right, is that you have a message that pertains to a patient. Before, we would send an email and then send a PID, and you had to look up that PID, which was incredibly cumbersome. Now, you go directly to the record that the message pertains to, so it’s a very efficient and good system.*

[Surgical physician 9]

However, some surgical physicians felt that the ease of sending chat messages had resulted in an overwhelming volume of communications, adding to the workload. They sometimes received messages regarding minor issues that could have been resolved without their intervention. Some also noted that it had become easier to access information regarding the care provided by health care professionals from other health care organizations that had also transitioned to the new EHR system. However, they still faced challenges when accessing information from organizations that had not adopted the new EHR system.

*I think the idea of having a shared system between primary health care, hospitals, and nursing homes is very good. The concept behind it is great, but the current situation is that many general practitioners don’t want, in fact, very few of them, if any at all, are using the new EHR. This poses a major problem because the feedback from general practitioners is that they are not receiving information from us […]. However, in some cases, where certain municipal institutions are using the new EHR, it works well. There, I can actually see the patient’s medication list and what they are actually using, so if everyone had used it and it had been a little bit more user-friendly, then I think it would have been a very good system.*

[Surgical physician 5]

Some informants also noted that communicating with patients through the patient health portal had become more straightforward. The patient portal referred to in this study is part of the new EHR system project, which was designed to enhance patient access to their medical records. However, some of them also pointed out that surgical physicians could not determine whether a message had been received or read by the patient.

*Yes, for those who have this patient portal, it’s really easy, such short messages, and you also get messages back, so communication between the patient and the physician, which we didn’t had before, works really well […].*

[Surgical physician 6]

#### Better Overview of Hospitalized Patients by Using the Timeline Function

Several informants emphasized the value of the timeline function for monitoring hospitalized patients. This function provided a visual representation of the patient’s condition, enabling surgical physicians to monitor the progression of diseases, particularly in cases of infections or chronic illnesses such as diabetes. The informants appreciated the ability to see the development day by day, which was considered important for treatment and decision-making.

*[…] there is a timeline function where you can plot in vital parameters, such as blood pressure and pulse, and then you get something like a graphical display thing. This allows you to monitor the infection, observe the levels of leukocytes, CRP, and temperature, as well as the medication they receive and the dosage, serum levels, concentration, and so on. It works quite well to track the progression of a disease and so on […].*

[Surgical physician 3]

### Mixed Methods Integration

The integration of quantitative and qualitative findings revealed both similarities and differences in physicians’ perceptions of the new EHR system (Table 2). Findings from both datasets were consistent regarding the presentation of medication lists and the EHR system’s ability to provide a summary view of patients’ health status. In the survey, most physicians expressed dissatisfaction with these aspects of the system, which aligned with interview accounts describing fragmented information, difficulties locating relevant patient data, duplicate information, and information overload. Differences between datasets regarding communication and cooperation between physicians and patients were observed. Although most survey respondents reported that the EHR system did not adequately support communication with patients, several surgical physicians interviewed in the qualitative component described positive experiences with specific functionalities, particularly the patient portal.

The qualitative findings also provided additional insights that were not captured by the survey items. In particular, physicians highlighted improved access to information from other health care organizations, more efficient communication between health care professionals through the chat function, and the usefulness of the timeline function for monitoring hospitalized patients.

**Table 2.** Joint display integrating quantitative and qualitative findings.

Meta-inference	Quantitative findings	Qualitative findings
Convergence: Both datasets indicated dissatisfaction with medication list presentation.	Only about 10% of physicians agreed that medication lists were presented clearly.	Surgical physicians described difficulty distinguishing preadmission medications from new medications and reported that medication lists were often difficult to interpret.
Convergence: Both datasets indicated difficulties with obtaining an overview of patients’ health status. The qualitative findings expanded the understanding of why physicians experienced difficulties obtaining a comprehensive summary view of patients’ health status.	Most physicians (>70%) disagreed that the new EHR^a^ system generated a useful summary view of patient health status.	Physicians described information overload, fragmented information, duplicated information, and extensive navigation between screens.
Divergence: The survey results indicated overall dissatisfaction, whereas interviews identified specific communication functions perceived positively.	Most physicians disagreed that the new EHR supports communication and cooperation between patients and clinicians.	Several physicians reported positive experiences with the patient portal and easier messaging with patients, although limitations remained.
Expansion: The qualitative findings identified benefits of the cross-institutional EHR that were not captured by the survey items.	Not assessed in the survey	Physicians reported improved access to information from municipalities, home care services, waiting lists, and image sharing capabilities.
Expansion: The qualitative findings provided additional insight into aspects of the EHR system that were not captured by the survey items.	Not assessed in the survey	Physicians highlighted timeline visualization as valuable for monitoring patient progress.

a EHR: electronic health record.

## Discussion

### Principal Findings

The findings from this study provide a nuanced understanding of physicians’ experiences with the newly implemented cross-institutional EHR system. The divergent perceptions regarding the quality, access, and presentation of patient data, as well as the EHR system’s effectiveness at supporting cooperation and communication with patients, underscore the complexity of integrating new technology into clinical practice. Although the quantitative data revealed substantial dissatisfaction among both surgical and nonsurgical physicians, the qualitative insights showed a more multifaceted picture that includes both positive and negative aspects of the EHR system. This duality in perceptions highlights the importance of examining both quantitative and qualitative data when evaluating the impact of EHR systems on clinical practice, in order to get a nuanced picture of users’ perceptions.

The quantitative data revealed that <10% of physicians agreed with how medication lists are presented in the new EHR system. Low satisfaction in this regard was also reported in the qualitative part of the study. Interestingly, some informants from the qualitative part of the study also revealed that, although entering medication information and performing medication reconciliation was cumbersome and difficult, once the process was completed, the final presentation of the medication list was clear and easy to understand. This juxtaposition suggests that improvements should focus not only on the final output—the presentation of medication lists—but also on streamlining the input processes that precede it. This also suggests that a hampered user experience during the input phase might impact the overall functionality and satisfaction with the output. A comprehensive study examining medication errors associated with health information technology (HIT) systems, drawing from patient safety reports across more than 595 health care facilities, found that, of the 2700 reports analyzed, 55.9% were related to HIT errors. Notably, 97.3% of these HIT-related errors stemmed from usability challenges, including difficulties with data entry, inadequate workflow support, and alerting issues [66].

An EHR system’s ability to provide a summary view of the patient’s health status is important for mitigating physicians’ cognitive load and improving clinical reasoning [67]. Clinical reasoning is defined as “the cognitive process that is necessary to evaluate and manage a patient’s medical problems” [68]. The quantitative findings from our study, however, indicate that more than 70% of physicians disagreed with the notion that the new EHR provided a comprehensive summary of a patient’s health status. The qualitative results further underscored this, as many informants expressed concerns about the overwhelming amount of information contained in the new system, noting that data often were scattered across various sections. This fragmentation necessitates multiple clicks to locate specific information. Additionally, the same information can appear in different sections, albeit in varying layouts, which further complicates the search process. These findings are consistent with previous research that noted the challenges of information overload [69,70] and data fragmentation [69,71] associated with EHR system use. Diamond et al [72] found that, following the EpicCare EHR implementation at 2 medical care centers, clinical staff reported lower perceptions of ease of use and cognitive support. The authors suggested that the introduction of new interfaces and workflows for accessing previously siloed data, among other factors, may have contributed to these perceptions. The sheer volume of data, along with its fragmentation and unwieldy presentation, impairs clinicians’ ability to effectively process information in a timely manner [73]. This challenge could extend the decision-making process and compromise the quality of the decisions made [69]. This could further elevate the probability of errors, such as overlooked test results; complicate the diagnostic procedure; and influence treatment decisions, thereby jeopardizing patient safety [70,74,75]. Indeed, the report on the new EHR system (examined in this study) from the National Audit Office of Norway (Riksrevisjonen) highlighted ongoing concerns among health care professionals about patient safety [76]. In 2023, 89.2% of physicians and 78.4% of nurses perceived the new system as a potential threat to patient safety. Although these worries were less prominent in 2024, they still remained significant, with 83.6% of physicians and 64.1% of nurses expressing similar concerns [76]. It is important to note that the survey did not specify that health care professionals perceived the new EHR as a threat to patient safety due to information overload and data fragmentation. The report further highlighted that patient safety was compromised because discharge summaries and referrals did not always reach their intended recipients within the treatment process, among other reasons [76]. This aligns with what our informant pointed out: a specific case where the patient was assessed twice because the physician’s previously written note was not visible in the EHR to health care professionals in the emergency department. Nevertheless, a later report by Vista Analyse [77] indicated that the quality of patient care returned to the same levels as before the implementation of the new EHR system and that there were no indicators suggesting a decline in patient safety. The reports also highlighted that several measures have been implemented to tackle the challenges identified, which were particularly high during the initial phase following the rollout. This underscores the complexity of deploying a cross-institutional EHR system and the fact that some issues will become apparent only after implementation, requiring time and targeted efforts to address them.

During our observation sessions and subsequent discussions with surgical physicians, it also became clear that the amount of information displayed on the screen can be overwhelming. Interestingly, although the new EHR system allows physicians to personalize certain aspects of the EHR, such as screen layouts, some physicians expressed that they had not received sufficient training or had enough time to fully explore and utilize this feature. This emphasizes the need for comprehensive training for users, along with adequate time dedicated to this training, so they can fully leverage the capabilities of the EHR system. The significance of training, particularly during the postimplementation phase, has been underscored in various studies [78-80]. Additionally, when considering the EHR system’s ability to present a summary of a patient’s health status, it is important to emphasize a key finding from our previous related work on physicians’ experiences with the new EHR: Some physicians harbor a distrust of the structured information found in EHR, such as patients’ Problem Lists, which are intended to aid with managing the patient’s health status [41]. This distrust stems from their awareness that many colleagues do not consistently document data in a structured format. Moreover, even when a Problem List is present, they often question whether it is up to date [41]. This issue might contribute to the fact that physicians disagreed that the new EHR system provided a summary view of the patient’s health status. This is an important factor, as data accuracy is influenced by the accuracy of documentation practices [81,82]. Furthermore, Miller and Sim [83] identified a link between better documentation by physicians and quality improvement, along with financial benefits.

Our study’s qualitative findings also revealed that the new EHR system had broadened access to patient health data. This includes better access to information from various health care organizations, such as home health care services, as well as more streamlined access to administrative data, including waiting lists. Miller and Sim [83] also reported comparable findings regarding enhanced access to patient health data through the use of EHR systems. The majority of our informants also emphasized that the timeline function offers a comprehensive overview of hospitalized patients, which is regarded as highly beneficial for monitoring their progress. Additionally, the new system promotes secure and efficient communication among health care staff through its chat function. These advantages underscore the value of an integrated EHR system that serves multiple health care organizations, including municipalities and hospitals, that encompass both primary and specialist health professionals. Moreover, some informants in the qualitative part of our study expressed that the patient portal facilitated easier communication with patients. However, this perspective was not mirrored in the quantitative findings, where 49.3% of surgical physicians and 62.9% of nonsurgical physicians disagreed with the assertion that the new EHR system enhances collaboration and communication between clinicians and patients. This discrepancy between qualitative and quantitative results underscores the necessity for further research in this area. Upon reviewing the existing literature on this topic, we found a comprehensive systematic review by Dendere et al [84] that highlighted the benefits of patient portals for identifying medical errors and improving medication adherence, as noted in 6 of the 58 studies included in their review. The review also emphasized enhanced communication between patients and health care providers, observed in 5 studies. However, it also pointed out potential drawbacks, such as increased anxiety in some patients [84].

Sherer et al [85] emphasized in their longitudinal qualitative study that, over time, users begin to appreciate how the EHR system enhances clinical information retrieval and improves care coordination as EHR data become more comprehensive and tailored to their needs. Although some of our informants echoed this sentiment, it was not a widespread view at the point of our data collection. Thus, evaluating the impact of a new EHR system over an extended period could offer valuable insights into its long-term effects. This is particularly important given that realizing the full potential of EHR systems requires changes and standardization in health care professionals’ workflows, a process that takes time. Sherer et al [85] noted that “users’ perspective on standardization evolves from initial concern about the unintended consequences of standardization to recognition of its importance and then finally to more active acceptance.” However, as Orlikowski [32] argued, structures can also evolve through practice and are influenced by user actions. Therefore, technology must possess sufficient flexibility to accommodate this adaptive process [32]. Moreover, Miller and Smith [83] emphasized that the advantages of adopting and using EHR systems in terms of quality improvement may not be immediate. Instead, these benefits could take several years to materialize as users become more proficient with the system. Therefore, it is reasonable to expect that, over time, physicians will increasingly embrace standardized work processes, including documentation practices, that could enhance data quality and interoperability. However, achieving these changes is challenging without coordinated efforts from the organization [85].

Our study’s findings reveal the complex interplay between technological factors—such as duplication of information across multiple locations within the EHR system—and social factors, including physicians’ unfamiliarity with the system and insufficient training. These factors could shape physicians’ use of the EHR system and their perceptions of it. Thus, ongoing assessment of EHR systems, considering both social and technological factors, will help identify challenges associated with EHR use. Such insights can guide EHR vendors and health care organizations in pinpointing key areas for improvement, ultimately enhancing usability and facilitating better integration of the system into the workflows of health care professionals.

### Limitations of the Study

The findings of this study should be viewed with certain limitations in mind. First, the qualitative results were derived from a relatively small sample size, comprising only 14 surgical physicians from a single department within the hospital. As such, these findings may not accurately represent the physicians’ perspectives in different clinical contexts. Second, the quantitative survey data relied on self-reported responses. Although we supplemented this with direct observations and individual interviews to address some of these limitations, it was not feasible to observe all physicians who participated in the survey. Additionally, we could not determine whether the surgical physicians involved in the qualitative part of the study also took part in the survey. This raises the possibility of selection bias, explaining why the perspectives of survey participants may differ from those of qualitative participants, as physicians’ viewpoints can vary depending on their working context. Moreover, the findings of this study should be interpreted in light of contextual factors that may have influenced participation and response. The implementation of the new EHR system was accompanied by substantial negative media coverage and resistance from staff, which may have introduced participation bias. Thus, we cannot ascertain whether the participants in the survey were more likely to engage due to a predisposition toward negative attitudes regarding the new EHR system or whether those more affected by the system were less able or willing to engage. Similarly, in the qualitative part of the study, we could not ascertain the extent to which participants’ attitudes toward the new EHR system influenced their engagement. Additionally, in such a context, participants may have been reluctant to express positive views due to perceived social or professional judgment. Nonetheless, several measures were taken to mitigate potential bias. Data were collected through individual face-to-face interviews rather than group discussions, thereby reducing peer influence. Also, the data collector (OG) had no prior relationship with the participants, limiting the risk of pre-existing bias. Combining direct observations with interview data allowed for triangulation, which further strengthened the credibility of the findings. Last, being preoccupied with a high workload due to patient treatment and learning to use a new EHR system may have further contributed to the low response rate.

### Conclusion

This study reveals a complex landscape regarding physicians’ experiences with the newly implemented cross-institutional EHR system. Physicians stressed the need for improved organization and presentation of patient health data while highlighting the positive impact of a cross-institutional EHR system on enhanced access to patient data. Although quantitative data showed that most physicians disagreed on whether the new EHR enhances cooperation and communication with patients, qualitative data highlighted positive experiences with the patient health portal for enhancing communication. Our findings suggest that adding a feature allowing users to confirm whether patients have received or read messages could further improve the usefulness of the patient portal. Notably, although the final output of medication lists was praised for clarity, the cumbersome input process could possibly detract from overall satisfaction, suggesting a need for enhancements in usability and training.

## Supplementary material

10.2196/90797 Multimedia Appendix 1 Semistructured interview guide.

10.2196/90797 Multimedia Appendix 2 Questionnaire.

10.2196/90797 Multimedia Appendix 3 Row counts and item-specific denominators for all National Usability-Focused Health Information System—Scale (NuHISS) items used in the analysis of this study.

10.2196/90797 Checklist 1 GRAMMS (Good Reporting of A Mixed Methods Study), SRQR (Standards for Reporting Qualitative Research), and STROBE (Strengthening the Reporting of Observational Studies in Epidemiology) checklists.

## Abbreviations

EHR: electronic health record
EPR: electronic patient record
GRAMMS: Good Reporting of A Mixed Methods Study
HIS: health information system
HIT: health information technology
NeRN: Nordic eHealth Research Network
NTNU: Norwegian University of Science and Technology
NuHISS: National Usability-Focused Health Information System—Scale
REK: Regional Committee for Ethics in Medical and Health Research Ethics
SRQR: Standards for Reporting Qualitative Research
STROBE: Strengthening the Reporting of Observational Studies in Epidemiology
STUNTH: The Study of New Technology and Health among Hospital employees
WHO: World Health Organization
