# Supplementary material for: Physicians' Perceptions of Information Quality and Access in a New Cross-Institutional Electronic Health Record (EHR) System: Mixed Methods Design
Source: JMIR Form Res. 2026 Oct 20;10:e90797. doi: 10.2196/90797 (PMC13683988; doi:10.2196/90797)
Supplement: Supplementary file 1 [file formative-v10-e90797-s001.pdf]

Supplementary Data 1: Semi-structured interview guide

1. How old are you?
2. When did you finish your medical education?
3. Specialization (if any)?
4. Can you describe your current position/role at the hospital?
5. Do you usually work in multiple healthcare facilities? For example, inpatient ward, emergency room, etc.
6. Previous experience with EHR (which types of EHR systems have you used before)?

Training in/experience with the new EHR system

7. How long have you been using the new EHR?
8. How often do you use the new EHR in your work?
9. Can you tell me a bit about the training you have received in the use of the new EHR?
10. How do you evaluate the training in the use of the EHR, particularly concerning clinical documentation and accessing patient data?
11. Are there aspects of the training that you believe could have been improved or done differently?

Clinical learning

12. What are your thoughts on accessing patient data in the new EHR system that has been entered by other healthcare professionals?
13. What do you think, is the patient’s current medication list presented in a clear format?
14. Does the EHR system generate a summary view (e.g., on a timeline) that helps to develop an overall picture of the patient's health status?
15. What do you think, does the new EHR system support co-operation and communication between clinicians and patients?
16. Does the new EHR system provide you with data and insights that you can use to learn from? For example, treatment effectiveness, tracking health outcomes
17. Have you ever modified your clinical practice based on insights or information acquired through the system?
18. Have you heard of Slicere Dicer? Do you use it? Any experiences with it?
19. How do you receive feedback on the work you do?
20. Do you use the system to find out the outcomes of the patients you have treated?

Thank you for your participation!
