# Supplementary material for: Physicians' Perceptions of Information Quality and Access in a New Cross-Institutional Electronic Health Record (EHR) System: Mixed Methods Design
Source: JMIR Form Res. 2026 Oct 20;10:e90797. doi: 10.2196/90797 (PMC13683988; doi:10.2196/90797)
Supplement: Supplementary file 2 [file formative-v10-e90797-s002.pdf]

Supplementary Data 2: Questionnaire

The patient's current medication list is presented in a clear format.

- Strongly agree
- Agree
- Neither/nor
- Disagree
- Strongly disagree
- Not relevant

The EHR system generates a summary view (e.g., on a timeline) that helps to develop an overall picture of the patients health status.

Strongly agree

- Agree
- Neither/nor
- Disagree
- Strongly disagree
- Not relevant

EHR systems support co-operation and communication between clinicians and patients

- Strongly agree
- Agree
- Neither/nor
- Disagree
- Strongly disagree
- Not relevant
