# Supplementary material for: Physicians' Perceptions of Information Quality and Access in a New Cross-Institutional Electronic Health Record (EHR) System: Mixed Methods Design
Source: JMIR Form Res. 2026 Oct 20;10:e90797. doi: 10.2196/90797 (PMC13683988; doi:10.2196/90797)
Supplement: Supplementary file 3 [file formative-v10-e90797-s003.docx]

**Table 1.** Physicians' perceptions of whether the patient's current medication list is presented in a clear format. Data are expressed as percentages with exact values and 95% confidence intervals.

| Surgical physicians (n = 71) | | | |
| --- | --- | --- | --- |
| **Response** | **n** | **%** | **95% CI** |
| Agree | 7 | 9.86% | 4.76 to 19.3 |
| Neither/nor | 7 | 9.86% | 4.76 to 19.3 |
| Disagree | 39 | 54.93% | 43.3 to 66.1 |
| Not relevant | 18 | 25.35% | 16.5 to 36.7 |
| Non-surgical physicians (n = 283) | | | |
| **Response** | **n** | **%** | **95% CI** |
| Agree | 26 | 9.19% | 6.3 to 13.2 |
| Neither/nor | 19 | 6.71% | 4.3 to 10.3 |
| Disagree | 238 | 84.10% | 79.3 to 87.9 |
| Not relevant | 0 | 0.00% | 0.0 to 1.3 |

**Table 2.** Physicians' perceptions of whether the new EHR (electronic health record) system generates a summary view that helps to develop an overall picture of the patient's health status. Data are expressed as percentages with exact values and 95% confidence intervals.

| Surgical physicians (n = 71) | | | |
| --- | --- | --- | --- |
| **Response** | **n** | **%** | **95% CI** |
| Agree | 4 | 5.6% | 8.5 to 3.5 |
| Neither/nor | 9 | 12.7% | 9.9 to 6 |
| Disagree | 54 | 76.1% | 8.5 to 11.3 |
| Not relevant | 4 | 5.6% | 8.5 to 3.5 |
| Non-surgical physicians (n = 283) | | | |
| **Response** | **n** | **%** | **95% CI** |
| Agree | 46 | 16.2% | 4.7 to 3.8 |
| Neither/nor | 35 | 12.4% | 4.4 to 3.4 |
| Disagree | 201 | 71% | 5 to 5.6 |
| Not relevant | 1 | 0.4% | 0.2 to 0.35 |

**Table 3.** Physicians' perceptions of whether the new EHR (electronic health record) system supports co-operation and communication between clinicians and patients. Data are expressed as percentages with exact values and 95% confidence intervals.

| Surgical physicians (n = 71) | | | |
| --- | --- | --- | --- |
| **Response** | **n** | **%** | **95% CI** |
| Agree | 7 | 9.8% | 9.44 to 5.1 |
| Neither/nor | 23 | 32.4% | 11.7 to 9.8 |
| Disagree | 35 | 49.3% | 11.5 to 11.4 |
| Not relevant | 6 | 8.5% | 9.1 to 4.7 |
| Non-surgical physicians (n = 283) | | | |
| **Response** | **n** | **%** | **95% CI** |
| Agree | 50 | 17.7% | 4.9 to 4.1 |
| Neither/nor | 50 | 17.7% | 4.9 to 4.1 |
| Disagree | 178 | 62.9% | 5.4 to 5.8 |
| Not relevant | 5 | 1.7% | 2.5 to 1 |
