# Supplementary material for: Physicians' Perceptions of Information Quality and Access in a New Cross-Institutional Electronic Health Record (EHR) System: Mixed Methods Design
Source: JMIR Form Res. 2026 Oct 20;10:e90797. doi: 10.2196/90797 (PMC13683988; doi:10.2196/90797)
Supplement: Supplementary file 4 [file formative-v10-e90797-s004.docx]

**Reporting Checklists**

Table 1. GRAMMS- Good Reporting of A Mixed Methods Study [1]

| **Guideline** | **Location in manuscript** |  |
| --- | --- | --- |
| Describe the justification for using a mixed methods approach to the research question | Methods - under Study Design pages 5-6 |  |
| Describe the design in terms of the purpose, priority and sequence of methods | Methods - under Study Design pages 5-6, and under Figure 1: Data collection process. |  |
| Describe each method in terms of sampling, data collection and analysis | Methods - under Qualitative data collection and study population pages 8-10; Methods - under Qualitative data analyses pages 10-11; Methods -under Quantitative data collection and study population, pages 12-13; and Methods - under Statistical Analysis, page 13. | |
| Describe where integration has occurred, how it has occurred and who has participated in it | Methods - under Study Design pages 5-6 | |
| Describe any limitation of one method associated with the present of the other method | Limitations of the study, pages 32-33 |  |
| Describe any insights gained from mixing or integrating methods | Results- under Mixed-methods integration; Table 2: Joint display integrating quantitative and qualitative findings; and under Discussion, pages 26-29 |  |

Table1. SRQR -Standards for reporting qualitative research [2]

| No | Topic | Location in manuscript |
| --- | --- | --- |
| Title and abstract | | |
| S1 | Title | Page 1 |
| S2 | Abstract | Page 2 |
| Introduction | | |
| S3 | Problem formulation | Page 3-4 |
| S4 | Purpose or research question | Page 3-4 |
| Methods | | |
| S5 | Qualitative approach and research paradigm | Page 4-6 and 8-11 |
| S6 | Research characteristics and reflexivity | Page 11 |
| S7 | Context | Page 7-8 |
| S8 | Sampling strategy | Page 8-10 |
| S9 | Ethical issue pertaining to human subjects | Page 34 |
| S10 | Data collection methods | Page 8-10 |
| S11 | Data collection instruments and technologies | Page 8-10 |
| S12 | Units of study | Page 9 |
| S13 | Data processing | Page 8-11 |
| S14 | Data analysis | Page 10-11 |
| S15 | Techniques to enhance trustworthiness | Page 10-11 |
| Results and findings | | |
| S16 | Synthesis and interpretation | Page 17-24 |
| S17 | Links to empirical data | Page 17-24 |
| Discussion | | |
| S18 | Integration with prior work, implications, transferability, and contribution (s) to the field | Page 26-31 |
| S19 | Limitations | Page 32-33 |
| Other | | |
| S20 | Conflict of interest | Page 35 |
| S21 | Funding | Page 36 |

Table 3. STROBE Statement—Checklist of items that should be included in reports of ***observational studies*** [3]

|  | Item No | Recommendation | Location in manuscript |
| --- | --- | --- | --- |
| **Title and abstract** | 1 | (*a*) Indicate the study’s design with a commonly used term in the title or the abstract | Page 1-2 |
|  |  | (*b*) Provide in the abstract an informative and balanced summary of what was done and what was found | Page 2 |
| Introduction | | | |
| Background/rationale | 2 | Explain the scientific background and rationale for the investigation being reported | Page 3-4 |
| Objectives | 3 | State specific objectives, including any prespecified hypotheses | Page 4 |
| Methods | | | |
| Study design | 4 | Present key elements of study design early in the paper | Page 5-6 |
| Setting | 5 | Describe the setting, locations, and relevant dates, including periods of recruitment, exposure, follow-up, and data collection | Page 7-8; Page 12-13 |
| Participants | 6 | (*a*) Give the eligibility criteria, and the sources and methods of selection of participants | Page 12-13 |
| Variables | 7 | Clearly define all outcomes, exposures, predictors, potential confounders, and effect modifiers. Give diagnostic criteria, if applicable | Pages 12-13 |
| Data sources/ measurement | 8* | For each variable of interest, give sources of data and details of methods of assessment (measurement). Describe comparability of assessment methods if there is more than one group | Page 12-13 |
| Bias | 9 | Describe any efforts to address potential sources of bias | Page 12-13 |
| Study size | 10 | Explain how the study size was arrived at | Page 12 |
| Quantitative variables | 11 | Explain how quantitative variables were handled in the analyses. If applicable, describe which groupings were chosen and why | Page 13 |
| Statistical methods | 12 | (*a*) Describe all statistical methods, including those used to control for confounding | Page 13 |
|  |  | (*b*) Describe any methods used to examine subgroups and interactions | Page 12-13 |
|  |  | (*c*) Explain how missing data were addressed | Page 13 |
|  |  | (*d*) If applicable, describe analytical methods taking account of sampling strategy | Not applicable |
|  |  | (*e*) Describe any sensitivity analyses | Not applicable |
| Results | | | |
| Participants | 13* | (a) Report numbers of individuals at each stage of study—eg numbers potentially eligible, examined for eligibility, confirmed eligible, included in the study, completing follow-up, and analysed | Page 14 |
|  |  | (b) Give reasons for non-participation at each stage | Page 12 |
|  |  | (c) Consider use of a flow diagram |  |
| Descriptive data | 14* | (a) Give characteristics of study participants (eg demographic, clinical, social) and information on exposures and potential confounders | Page 14; Table 1 |
|  |  | (b) Indicate number of participants with missing data for each variable of interest | Page 13; Page 14; Supplementary Data 3 |
| Outcome data | 15* | Report numbers of outcome events or summary measures | Page 14-17; Figures 2-4 |
| Main results | 16 | (*a*) Give unadjusted estimates and, if applicable, confounder-adjusted estimates and their precision (eg, 95% confidence interval). Make clear which confounders were adjusted for and why they were included | Page 14-17; Figures 2-4; Supplementary Data 3 |
|  |  | (*b*) Report category boundaries when continuous variables were categorized | Page 14 |
|  |  | (*c*) If relevant, consider translating estimates of relative risk into absolute risk for a meaningful time period | Not applicable |
| Other analyses | 17 | Report other analyses done—eg analyses of subgroups and interactions, and sensitivity analyses | Page 24-26; Table 2 |
| Discussion | | | |
| Key results | 18 | Summarise key results with reference to study objectives | Page 26-31 |
| Limitations | 19 | Discuss limitations of the study, taking into account sources of potential bias or imprecision. Discuss both direction and magnitude of any potential bias | Page 32-33 |
| Interpretation | 20 | Give a cautious overall interpretation of results considering objectives, limitations, multiplicity of analyses, results from similar studies, and other relevant evidence | Page 26-33 |
| Generalisability | 21 | Discuss the generalisability (external validity) of the study results | Page 32-33 |
| Other information | | | |
| Funding | 22 | Give the source of funding and the role of the funders for the present study and, if applicable, for the original study on which the present article is based | Page 36 |

*Give information separately for exposed and unexposed groups.

**Note:** An Explanation and Elaboration article discusses each checklist item and gives methodological background and published examples of transparent reporting. The STROBE checklist is best used in conjunction with this article (freely available on the Web sites of PLoS Medicine at http://www.plosmedicine.org/, Annals of Internal Medicine at http://www.annals.org/, and Epidemiology at http://www.epidem.com/). Information on the STROBE Initiative is available at www.strobe-statement.org.

1.	O’cathain A, Murphy E, Nicholl J. The Quality of Mixed Methods Studies in Health Services Research. J Health Serv Res Policy 2008 Apr;13(2):92–98. doi: 10.1258/jhsrp.2007.007074

2.	O’Brien BC, Harris IB, Beckman TJ, Reed DA, Cook DA. Standards for Reporting Qualitative Research: A Synthesis of Recommendations. Academic Medicine 2014 Sept;89(9):1245–1251. doi: 10.1097/ACM.0000000000000388

3.	von Elm E, Altman DG, Egger M, Pocock SJ, Gøtzsche PC, Vandenbroucke JP. The Strengthening the Reporting of Observational Studies in Epidemiology (STROBE) statement: guidelines for reporting observational studies. The Lancet Elsevier; 2007 Oct 20;370(9596):1453–1457. doi: 10.1016/S0140-6736(07)61602-X
